# Supplementary material for: Nanopore Sequencing-Driven Mapping of Antimicrobial Resistance Genes in Selected Escherichia coli Isolates from Pigs and Poultry Layers in Nigeria
Source: Antibiotics (Basel). 2025 Aug 14;14(8):827. doi: 10.3390/antibiotics14080827 (PMC12382671; doi:10.3390/antibiotics14080827)
Supplement: Supplementary file 1 [file antibiotics-14-00827-s001.zip › antibiotics-3721966-supplementary.pdf]

Supplementary Table 1. Characterization of *E. coli* isolates: sequencing metrics, resistance, virulence and plasmid content.

ID isolates	Number of Reads	Number of nucleotidess	Average sequence length	Specie	Origin	Antibiotic resistance pattern	Antimicrobial resistance genes	Virulence factor genes	Replicon plasmidic
118p1	404	735850	1821	*Escherichia coli*	pigs	P	*EvgS, baeS, baeR*	*gspL, tssL, espX5, vgrG/tssI, tssG, tssC, tssF, fha, tssJ, tssC, tssB, hcp2/tssD2*	
023 2	624	307719	493	*Escherichia coli*	pigs	CIP, TE, AMP, TIC, DO, P	*acrD, bcr*		
115p2	2322	3422193	1474	*Escherichia coli*	pigs	P	*mdtB, mdtM, CRP, mdtA, gadX, PmrC, cpxA, mdtE, acrE, patA, emrY, acrS, emrK, evgA, wzx-Onovel9*	*gspJ, fimI, fimC, gspI, tssA, fepG, entD, fimB, gspK, tssB, fimE, tssC*	
083p1	1865	3494688	1874	*Escherichia coli*	pigs	P	*mdtE, mdtA, emrR, acrD, H-NS, acrF, emrD, mdtF, acrS, msbA, mdtM, mdtP, baeS, cpxA, blaEC-18, CRP, gadX, acrE, PmrC, bacA*	*espX1, gspM, fimB, entD, yagW/ecpD, fimF, fimE, yagX/ecpC, fimH, fimG*	
005 2	283	422137	1492	*Escherichia coli*	pigs	CIP, P	*bacA*	*gspL, tssC, tssJ, hcp1/tssD1, tssC*	
063 3	3094	4865653	1573	*Escherichia coli*	pigs	CIP, TE, AMP, FEP, KZ, TIC, ATM, DO, P	*tet(A), baeS, evgA, emrY, PmrC, patA, emrK, acrD, CRP, bacA, mdtF, aph(6)-Id, emrB, msbA, cpxA, emrD, sul2, mdtH, mdtO, kdpE, mdtN, aph(3'')-Ib*	*gspI, fes, tssM, fepD, gspL, fepG, fepC, gspF, fimD, gspM*	IncHI1B(R27)_1_R27
114p1	2870	4562194	1590	*Escherichia coli*	pigs	CIP, P	*mdtP, evgA, emrB, mdtO, emrA, emrD, kdpE, cpxA, emrR, mdtF, mdtG, mdtA, mdtE, acrF, acrE, wzy-O8, H-NS, gadX, PmrC, wzx-O8*	*tssA, fepC, tssJ, hcp1/tssD1, fha, fepB, yagY/ecpB, yagX/ecpC, hcp2/tssD2, fimF, gspJ, ykgK/ecpR, tssM, gspC, gspL, entB, gspK, yagZ/ecpA, yagW/ecpD, tssA, gspH, tssB*	

36p2	1958	3295692	1683	*Escherichia coli*	pigs	CIP, TE, AMP, KZ, TIC, P	*mdtN, emrA, acrD, CRP, mdtB, blaEC-18, mdtO, cpxA, mdtP, emrR, mdtA, fliC-H20, emrB, evgA, Escherichia_coli_acrA*	*fimH, yagX/ecpC, ykgK/ecpR, yagY/ecpB*	ColpVC_1
101p1	4389	7069968	1611	*Escherichia coli*	pigs	CIP, CTX, CAZ, TE, AMP, KZ, TIC, DO, P	*marA, emrA, yojI, acrE, mdtE, emrD, mdf(A), mdtP, acrS, qnrS1, mdtH, PmrC, bacA, emrB, fliC-H5, mdtN, mdtG, Escherichia_coli_ampC, wzy-O124var1, evgS, qnrS15, cmlA1, qnrS10, gadX, tolC, emrK, aadA2, evgA, sul3, dfrA12*	*fepB, entD, espX1, yagV/ecpE, espL4, entC, yagZ/ecpA, ykgK/ecpR, espX4, entF, fes, espX5, fepA, yagY/ecpB, fimI, espL1, fimE*	ColpVC_1
69 3	1529	3962078	2591	*Escherichia coli*	chicken	CIP, AMC, AMP, TIC, DO, P, C	*mdtC, baeS, baeR, wzm-O9, emrD, mdtM, acrD, mdtH, msbA, mdtB, dfrA12, aadA2, CRP, evgS, tolC, acrS, kdpE, cmlA1, ant(3'')-Ia, patA, sul3, PmrC, fllA-H44*	*vgrG/tssI, fimB, fepB, entF, hcp1/tssD1, yagV/ecpE, fes, fepC, fepG, tssB, fha, tssG, yagW/ecpD, tssC, tssC, tssF, hcp2/tssD2*	

AMP: Ampicillin ; ATM: Aztreonam; CAZ: Ceftazidim; CTX: Cefotaxim; FEP: Cefepim; KZ: Cephazolin; CIP: Ciprofloxacin ; DO: Doxycyclin; TE: Tetracyclin; P: Penicillin; TIC: Ticarcillin

Supplementary Table 2. Distribution of the ARGs depicting the drug family, antibiotics and number of *E. coli* isolates harboring the resistant genes.

Drug family	Antibiotics	ARGs Name	Resistance Mechanism	Number of ARGs	ARGs in Command line, Number of isolate (N)	ARGs in EPI2ME, Number of isolate (N)
Fluoroquinolone antibiotic	Norfloxacin, Ciprofloxacin	*emrA*	antibiotic efflux	25	03	
		emrR	antibiotic efflux		04	
		emrB	antibiotic efflux		04	02
		mdtH	antibiotic efflux		03	
		evgA	antibiotic efflux		04	02
		Escherichia_acrA	antibiotic efflux		01	
		mdtM	antibiotic efflux		04	
		gadX	antibiotic efflux		05	02
		mdtE	antibiotic efflux		04	
		acrE	antibiotic efflux		04	
		acrS	antibiotic efflux		04	
		H-NS	antibiotic efflux		03	01
		mdtF	antibiotic efflux		03	
		acrB	antibiotic efflux		01	
		CRP	antibiotic efflux		04	
		marA	reduced permeability to antibiotic, antibiotic efflux		02	
		tolC	antibiotic efflux		03	01
		acrF	antibiotic efflux		03	02
		EvgS	antibiotic efflux		03	02
		qnrS1	antibiotic target protection		01	01
		qnrS15	antibiotic target protection		01	

		qnrS10	antibiotic target protection		01	
		Enterobacter_cloacae_acrA	antibiotic efflux		01	
		oqxB	antibiotic efflux		01	01
Aminoglycoside antibiotic	Gentamicin, Streptomycin, Tobramycin, Amikacin	*acrD*	antibiotic efflux	08	05	01
		cpxA	antibiotic efflux		04	01
		baeS	antibiotic efflux		04	01
		tolC	antibiotic efflux		03	01
		baeR	antibiotic efflux		03	
		kdpE	antibiotic efflux		02	
		aadA2	antibiotic inactivation		02	01
		ant(3′′)-Ia	antibiotic inactivation		01	
Penam	Ticarcillin, Piperacillin-Tozobactam, Ampicillin	*evgA*	antibiotic efflux	15	04	02
		gadX	antibiotic efflux		05	02
		mdtE	antibiotic efflux		04	
		acre	antibiotic efflux		04	
		acrS	antibiotic efflux		04	
		H-NS	antibiotic efflux		03	01
		mdtF	antibiotic efflux		03	
		acrB	antibiotic efflux		01	
		CRP	antibiotic efflux		04	
		marA	reduced permeability to antibiotic, antibiotic efflux		02	
		tolC	antibiotic efflux		03	01
		acrF	antibiotic efflux		03	02
		EvgS	antibiotic efflux		03	02
		Escherichia_coli_ampC	antibiotic inactivation		01	
		Enterobacter_cloacae_acrA	antibiotic efflux		01	
Phenicol antibiotic	Chloramphenicol	*Escherichia_coli_acrA*	antibiotic efflux	09	01	
		mdtM	antibiotic efflux		04	
		acrS	antibiotic efflux		04	

		acrB	antibiotic efflux		01	
		marA	reduced permeability to antibiotic, antibiotic efflux		02	
		tolC	antibiotic efflux		03	01
		emrD	antibiotic efflux		05	
		cmlA1	antibiotic efflux		02	
		Enterobacter_cloacae_acrA	antibiotic efflux		01	
Cephalosporin	Cephazolin, Cefotaxime, Cefepime, ceftazidime, ceftriaxone	*Escherichia_coli_acrA*	antibiotic efflux	10	01	
		H-NS	antibiotic efflux		03	01
		marA	reduced permeability to antibiotic, antibiotic efflux		02	
		tolC	antibiotic efflux		03	01
		acrF	antibiotic efflux		03	02
		Escherichia_coli_ampC	antibiotic inactivation		01	
		Enterobacter_cloacae_acrA	antibiotic efflux		01	
		acrE	antibiotic efflux		04	
		acrS	antibiotic efflux		04	
		acrB	antibiotic efflux		01	
Cephamycin	Cefoxitin	*acre*	antibiotic efflux	05	04	
		acrS	antibiotic efflux		04	
		H-NS	antibiotic efflux		03	01
		tolC	antibiotic efflux		03	01
		acrF	antibiotic efflux		03	02
Tetracycline antibiotic	Tetracycline, Doxycycline	*emrY*	antibiotic efflux	13	02	01
		emrK	antibiotic efflux		02	
		evgA	antibiotic efflux		04	02
		Escherichia_coli_acrA	antibiotic efflux		01	
		acrS	antibiotic efflux		01	
		H-NS	antibiotic efflux		03	01
		acrB	antibiotic efflux		01	

		marA	reduced permeability to antibiotic, antibiotic efflux		02	
		tolC	antibiotic efflux		03	01
		mdf(A)	antibiotic efflux		02	
		EvgS	antibiotic efflux		03	02
		Enterobacter_cloacae_acrA	antibiotic efflux		01	
		oqxB	antibiotic efflux		01	01
Phosphonic acid antibiotic	Fosfomycin	*mdtG*	antibiotic efflux	01	03	02
Monobactam	Aztreonam	*marA*	reduced permeability to antibiotic, antibiotic efflux	02	02	
		MIR-14	antibiotic inactivation		01	
Carbapenem	Ertapenem, Imipenem	*marA*	reduced permeability to antibiotic, antibiotic efflux	02	02	
		tolC	antibiotic efflux		03	01
Sulfonamide antibiotic	Trimethoprim-Sulfamethoxazole	*Sul3*	antibiotic target replacement	02	02	02
		dfrA12	antibiotic target replacement		02	
Beta-lactam	Amoxicillin-Clavulanate, Penicillin	*blaEc15*		03	01	
		blaEc18			03	
		blaACT58			01	
